# Quantum-like Cognition and Decision-Making: Interpretation of Phases in Quantum-like Superposition

**DOI:** 10.3390/e28020134

**Published:** 2026-01-23

**Authors:** Andrei Khrennikov

**Affiliations:** Center for Mathematical Modeling in Physics and Cognitive Sciences, Linnaeus University, SE-351 95 Vaxjoe, Sweden; andrei.khrennikov@lnu.se

**Keywords:** quantum-like cognition and decision-making, quantum-like modeling, phases in quantum-like superposition, phases of random oscillations, neuronal circuits in the brain

## Abstract

This paper addresses a central conceptual challenge in *Quantum-like Cognition and Decision-Making (QCDM)* and the broader research program of *Quantum-like Modeling (QLM)*: the interpretation of phases in quantum-like state superpositions. In QLM, system states are represented by normalized vectors in a complex Hilbert space, $|\psi \rangle =\sum_{k}X_{k}\;|k\rangle$, where the squared amplitudes $P_{k}={\left|X_{k}\right|}^{2}$ are outcome probabilities. However, the meaning of the phase factors $e^{i\phi_{k}}$ in the coefficients $X_{k}=P_{k}\;e^{i\phi_{k}}$ has remained elusive, often treating them as purely phenomenological parameters. This practice, while successful in describing cognitive interference effects (the “interference of the mind”), has drawn criticism for expanding the model’s parameter space without a clear physical or cognitive underpinning. Building on a recent framework that connects QCDM to neuronal network activity, we propose a concrete interpretation. We argue that the phases in quantum-like superpositions correspond directly to the phases of random oscillations generated by neuronal circuits in the brain. This interpretation not only provides a natural, non-phenomenological basis for phase parameters within QCDM but also helps to bridge the gap between quantum-like models and classical neurocognitive frameworks, offering a consistent physical analogy for the descriptive power of QLM.

## 1. Introduction

*Quantum-like Cognition and Decision-Making*(QCDM) belongs to the broader research program of *Quantum-like Modeling* (QLM), which applies the methodology and formalism of quantum theory outside physics, such as in biology, genetics, cognition, social and political sciences, economics, and finance. (Extended lists of related publications can be found in recent monographs [1,2,3] and reviews [4,5]; please also see the bibliography in [6]).

We want to emphasize that QLCD is fundamentally different from approaches that attempt to reduce mental processes to genuine quantum physical phenomena in the brain (see Umezawa and Vitiello [7,8,9], Penrose [10,11], Hameroff [12]; please also see [13,14,15,16,17,18]).

The terminology can be confusing. Some researchers, such as Aerts et al. [19,20] and Busemeyer and Bruza [2], use the term *“quantum cognition”* when describing what we call *quantum-like cognition*. At the same time, Hameroff [18] and Fisher [21] use the same phrase to refer to truly quantum physical processes as part of the quantum brain project. To avoid ambiguity, we use the term *quantum-like cognition* consistently. This paper is dedicated exclusively to this approach and is entirely separate from the quantum brain research program.

In QLM, the states of systems are represented either as pure quantum states, i.e., normalized vectors in a complex Hilbert space H, or more generally as mixed states described by density operators. Pure states are complex-valued superpositions of the form

(1) $$|\psi \rangle =\sum_{k}X_{k}\;|k\rangle ,\;\sum_{k}{\left|X_{k}\right|}^{2}=1,\;X_{k}\in C,$$

where {|k〉} is an orthonormal basis of H. The squared amplitudes $P_{k}={\left|X_{k}\right|}^{2}$ represent probabilities of outcomes for an observable diagonal on this basis. However, the interpretation of the phases in the expansion coefficients,

$$X_{k}=P_{k}\;e^{i\phi_{k}},$$

has remained unclear. In QCDM, such phases are expected to be related to the functioning of neuronal circuits, yet the nature of this coupling has not been well understood.

In most applications of QCDM, and of QLM more broadly, one works within a phenomenological framework: phases are treated as additional parameters of a probabilistic model. From a phenomenological standpoint, introducing such parameters helps to explain the descriptive power of QLM. Nevertheless, this practice has been criticized, especially within the numerical simulation community, on the grounds that expanding the parameter space trivially increases modeling capacity. This criticism is generally unfounded: phase parameters are not introduced ad hoc but arise naturally and consistently within quantum formalism. Still, QLM lacks a fully articulated interpretation of phases in quantum-like superpositions. In QCDM, these superpositions are central, especially in modeling cognitive interference effects—the so-called “interference of the mind” [22].

In [23] (please also see [6,24]), we took a step toward connecting QCDM with the functioning of neuronal networks in the brain (cf. [25,26,27,28,29,30,31]). Building on that framework, the present paper provides an interpretation of phases in quantum-like superpositions used in QCDM. We argue that these phases correspond to the phases of random oscillations generated by neuronal circuits or more generally by complex systems [32]. This interpretation applies not only to QCDM but more broadly to QLM. Our focus on QCDM stems from the clear cognitive meaning of random oscillatory activity in the brain and from the need to bring quantum-like models closer to classical neurocognitive frameworks.

## 2. Coupling Between Oscillatory Neuronal and Quantum-like Cognitive Models

In [23], we explored so-called Prequantum Classical Statistical Field Theory (PCSFT), which was developed in a series of works to show that the hidden variable project for quantum theory can be realized, with a proper modification [33].

To simplify considerations, everywhere below we assume that all classical random variables have zero mean values and finite second moments:

(2) $$E\left[\xi \right]=0,E[|\xi {|}^{2}]<\infty$$

If the mean value is not zero, we can always subtract it from a random variable.

### 2.1. Oscillatory Network (System *S*)

We consider a dynamic ensemble (or network) *S* where the constituent elements are coupled oscillators. Each such element models a *neuronal microcircuit* composed of recurrently interconnected neuron populations. These oscillatory neuronal circuits are hypothesized to serve as the fundamental units of information processing within the system.

It is crucial to note that the term “coupling” does not exclusively denote physical connections via axons and dendrites. For instance, in systems involving *electromagnetic oscillators* (see [33]), the coupling mechanism can be mediated by *electromagnetic fields*.

### 2.2. Classical Dynamics in Phase Space

In a basic (yet highly generalizable) mathematical framework, the oscillatory motion within each element is governed by a pair of conjugate variables: a generalized position $q_{j}=q_{j}\left(t\right)$ and its canonical momentum $p_{j}\left(t\right)={\dot{q}}_{j}\left(t\right)$ for j=1,…,N. Here, *N* denotes the *cardinality* (number) of these elementary circuits (oscillators) constituting the network *S*.

We define the configuration space as $Q=R^{N}$ and the momentum space as $P=R^{N}$. The total classical phase space of the composite system is then the direct product $X=Q\times P=R^{2N}$. The instantaneous *classical state* of the network *S* is specified by a point x=(q,p)∈X, where $q=(q_{1},\ldots ,q_{N})$ and $p=(p_{1},\ldots ,p_{N})$. This 2N-dimensional phase space *X* is naturally endowed with the following standard Euclidean scalar product:

$$\left(x,y\right)=\left(q_{x},q_{y}\right)+\left(p_{x},p_{y}\right)=\sum_{j}\left(q_{xj}q_{yj}+p_{xj}p_{yj}\right).$$

### 2.3. Complex Hilbert Space Representation (H)

To transition towards a QL representation within a complex Hilbert space, we introduce a set of complex-valued variables (often referred to as annihilation/creation-like variables) (cf. [34,35]):

$$z_{j}=\frac{1}{\sqrt{2}}\left(q_{j}+ip_{j}\right)$$

Consequently, at any moment in time, the network state is fully described by the complex vector $z=(z_{1},\ldots ,z_{N})$.

When the index *j* is treated as a continuous spatial variable, e.g., $x\in R^{3}$, the description encompasses distributed field systems. For electromagnetic oscillators, the canonical variables are the electric and magnetic field components q(x)=E(x) and p(x)=B(x). In this case, the complex field Z(x)=E(x)+iB(x) corresponds to the Riemann–Silberstein representation of the electromagnetic field [33].

This mapping achieves the *complexification of the classical phase space* X=Q×P and allows us to explore the corresponding complex Hilbert space H, which is constructed as the formal direct sum as follows:

$$H=Q⊕iP=C^{N}$$

This space is equipped with the standard Hermitian inner product as follows:

$$\langle u|v\rangle =\sum_{j}u_{j}{\bar{v}}_{j}$$

The associated norm is defined by the square of the magnitude as follows:

$${∥z∥}^{2}=\langle z|z\rangle =\sum_{j}{\left|z_{j}\right|}^{2}.$$

The distinct oscillatory nodes of the network naturally define the *canonical orthonormal basis* $\left(e_{j}=|j\rangle ,j=1,2,\ldots ,N\right)$ spanning H. Finally, transformations that are *symplectically linear* in the classical phase space *X* are mirrored by the action of *C-linear operators* in the complex Hilbert space H.

### 2.4. Quantum-like State Defined by Covariance

The QL state of the dynamical network *S* is mathematically encapsulated by the complex covariance matrix *C* of the underlying classical random complex vector $z\left(\omega \right)=(z_{1}\left(\omega \right),\ldots ,z_{N}\left(\omega \right),$ where ω is a random parameter. The entries of this matrix are defined by the expected values of the products of coordinates of this random vector, $z_{k}\left(\omega \right){\bar{z}}_{m}\left(\omega \right)$:

(3) $$C=\left(c_{km}\right),\;c_{km}=E\left[z_{k}{\bar{z}}_{m}\right].$$

The resulting covariance matrix *C* possesses two fundamental algebraic properties:

- It is Hermitian, satisfying $C^{*}=C$, which implies that the components are conjugate symmetric: $c_{ij}=\bar{c_{ji}}$.
- It is Positive Semidefinite, denoted as C≥0, meaning that the quadratic form 〈z|C|z〉≥0 holds for any vector $z\in C^{N}$.

We observe that these two properties are also the defining characteristics of density matrices in standard quantum theory. The only difference between covariance and density matrices is that a density matrix has trace one.

### 2.5. Normalization to a Density Matrix

We now normalize the covariance matrix *C* to yield the QL state ρ:

(4) $$C⟶\rho =F_{s}\left(C\right)=\frac{C}{\mathrm{Tr}(C)}$$

(The subscript *s* denotes “state”).

By construction, ρ has a unit trace (Tr(ρ)=1) and, therefore, serves as a density matrix. This establishes a profound connection: the random classical states of the network *S* of interacting oscillators can be formally mapped onto quantum states. The mapping $F_{s}$ is inherently non-injective; specifically, scaling the covariance matrix *C* by any positive scalar factor *c* results in the identical QL state ρ.

In this paper, we do not consider composite systems and, hence, the generation of entangled states from classical oscillations. In PCSFT, we can model even composite systems and their entangled states [33]. However, the construction is rather complicated, and in this paper, we prefer to clarify the origin of state’s phase for non-composite systems.

### 2.6. Cognitive Interpretation and Ambiguities

In [6], we speculate regarding the cognitive significance of covariance operators within QLCDM:

**Speculation** **1.** *The belief states (or mental states) relevant to decision-making, as modeled via QCDM, are coupled to underlying neurophysiological processes in the brain through the correspondence established by Equation (4). Within the brain, these beliefs are hypothesized to be represented by the correlations established between oscillating neuronal circuits that form complex functional networks. To process information in the abstract framework (such as concepts, relational semantics, and combinatorial representations), the brain utilizes the QL representation in the complex Hilbert space H, because this formalism naturally supports contextuality and the superposition of incompatible cognitive alternatives.*

Generally, a covariance matrix does not uniquely specify the underlying random vector. Consequently, the QL representation inherently offers a coarse-grained perspective on the microscopic behavior of the random oscillators within the neuronal networks. While hidden variables (the individual points in the oscillatory phase space) undeniably exist, they are rendered invisible in the aggregate QL description. Hence, QCDM operates at the meta-level, and the brain uses the formal representation of neuronal activity in terms of density matrices.

## 3. From Classical Correlations to Phases in Quantum States

### 3.1. Phases ϕ=0,π – Perfect (Anti-)Correlations

We start with an analysis of qubit states,

(5) $$|\psi \rangle =r_{0}|0\rangle +e^{i\phi }r_{1}|1\rangle ,$$

where $r_{0},r_{1}>0,r_{0}^{2}+r_{1}^{2}=1,\phi \in \left[0,2\pi \right).$

Consider the phases ϕ=0 and ϕ=π, so the states

(6) $$|\psi_{\pm }\rangle =r_{0}|0\rangle \pm r_{1}|1\rangle .$$

The corresponding density operators have the following form:

(7) $$\rho_{\pm }=|\psi_{\pm }\rangle \langle \psi_{\pm }\rangle |=r_{0}^{2}\left|0\rangle \langle 0\right|\pm r_{0}r_{1}(|0\rangle \langle 1|+|1\rangle \langle 0|)+r_{1}^{2}|1\rangle \langle 1|.$$

Now consider their classical preimages, covariance operators $C_{+}$ and $C_{-}$. In the qubit basis (|0〉,|1〉), they are given by the covariance matrices $C_{+}$ and $C_{-}$ of two real-valued random vectors, $X_{+}=\left(X_{0+},X_{1+}\right)$ and $X_{-}=\left(X_{0-},X_{1-}\right),$

(8) $$C_{+}=\left(c_{km+}=E\left[X_{k+}X_{m+}\right]\right),C_{-}=\left(c_{km-}=E\left[X_{k-}X_{m-}\right]\right)$$

leading to the operators

(9) $$C_{+}=\sum_{km}c_{km+}|k\rangle \langle m|,C_{-}=\sum_{km}c_{km-}|k\rangle \langle m|.$$

To make consideration clearer, assume that $\mathrm{Tr}C_{\pm }=1.$ Then, classical→quantum mapping (4) leads to the equalities

(10) $$c_{km\pm }=\pm r_{k}r_{m}.$$

So, phases ϕ=0 and ϕ=π correspond to correlations and anti-correlations, respectively, between the coordinates of random vectors behind the quantum(-like) states. For states $|\psi_{\pm }\rangle =[|0\rangle \pm |1\rangle ]/\sqrt{2}$, we obtain perfect correlations and anti-correlations, respectively:

(11) $$\mathrm{Corr}\left(X_{0\pm },X_{1\pm }\right)=\frac{E[X_{0\pm }X_{1\pm }]}{\sqrt{E\left[X_{0\pm }^{2}\right]E\left[X_{1\pm }^{2}\right]}}=\pm 1,$$

Then, they are coupled as

(12) $$X_{1\pm }\left(\omega \right)=\pm X_{0\pm }\left(\omega \right)$$

almost everywhere. (Here, ω is a random parameter.) They can be combined in the H-valued random vectors $\phi_{\pm }\left(\omega \right)=\xi \left(\omega \right)|\psi_{\pm }\rangle$ (almost everywhere), where ξ=ξ(ω) is a complex-valued random variable such that E[ξ]=0 and $E[|\xi {|}^{2}]=1.$

In the oscillatory cognition model behind QCDM, the quantum-like states $|\psi_{\pm }\rangle$ are generated by two neuronal circuits with outputs connected via (12). This represents the perfect (anti-)synchronization of neurons.

Thus, the meaning of superpostions with the phases ϕ=0,π was clarified.

We find that classical random variables describing the functioning of neuronal circuits behind quantum-like superpositions are not uniquely determined, as a variety of classical random vectors with perfectly (anti-)correlated coordinates generate the same states $|\psi_{\pm }\rangle .$

Take now the state $|\psi \rangle =\frac{1}{\sqrt{n}}\sum_{k=1}^{n}|k\rangle .$ So, $\rho =\left|\psi \rangle \langle \psi \right|=\frac{1}{n}\left(\begin{array}{ccccc} 1 & 1 & 1 & \cdots & 1\\ 1 & 1 & 1 & \cdots & 1\\ 1 & 1 & 1 & \cdots & 1\\ \vdots & \vdots & \vdots & \ddots & \vdots \\ 1 & 1 & 1 & \cdots & 1 \end{array}\right)$ Consider a random vector $X=(X_{1},\ldots ,X_{n})$ with the covariance matrix C=ρ, and then *X* has the form

X(ω)=ξ(ω)|ψ〉,

so $X_{i}\left(\omega \right)=\frac{\xi (\omega )}{\sqrt{n}}\;a.s.\;\mathrm{for}\;i=1,\ldots ,n,$ (almost everywhere). All components are equal almost surely (perfectly correlated). At the neuronal level, we have *n* synchronized circuits.

Let us start another way around. Start with a random vector $W=\left(X_{1},\ldots ,X_{k},Y_{1},\ldots ,Y_{m}\right)\in R^{k+m}$ (with zero mean), in which the following conditions are true almost everywhere:

- $X_{1}=\cdots =X_{k}=:X$ (perfectly correlated);
- $Y_{1}=\cdots =Y_{m}=:Y$ (perfectly correlated);
- *X* and *Y* are perfectly anti-correlated: Y=−X.

Let n=k+m. The covariance matrix of *W* is $C=\mathrm{Cov}\left[W\right]=\frac{1}{n}\left(\begin{array}{cc} J_{k} & -1_{k\times m}\\ -1_{m\times k} & J_{m} \end{array}\right),$ where $J_{k}=1_{k}1_{k}^{T}$, $J_{m}=1_{m}1_{m}^{T}$, and $1_{k\times m}=1_{k}1_{m}^{T}$ are all-one matrices.

In the oscillatory cognition model behind QCDM, we have two groups of neuronal circuits, and in each group, outputs are synchronized with synchronized cross-anti-correlations between the groups.

Since the covariance matrix *C* is rank-1 and positive semidefinite, it can be written as the density matrix of a pure state: $|\psi \rangle =\frac{1}{\sqrt{n}}\left(\begin{array}{c} 1\\ \vdots \\ 1\\ -1\\ \vdots \\ -1 \end{array}\right),$ where the first *k* entries are +1 (corresponding to *X* variables) and the last *m* entries are −1 (corresponding to *Y* variables).

### 3.2. Phase ϕ=π/2

Now we analyze phases which are different from ϕ=0,π. Consider a particular example, ϕ=π/2, and analyze one of the Bell states, namely the state

(13) $$|\psi \rangle =[|0\rangle +i|1\rangle ]/\sqrt{2},$$

and its density operator

(14) $$\rho =\left|\psi \rangle \langle \psi \right|=\frac{1}{2}[|0\rangle \langle 0|-i|0\rangle \langle 1|+i|1\rangle \langle 0|+|1\rangle \langle 1|].$$

Consider the complex random vector $X=(X_{0},X_{1}),$ where $X_{k}=Q_{k}+iP_{k}.$ We have

$$c_{01}=E\left[X_{0}\bar{X_{1}}\right]=E\left[Q_{0}Q_{1}+P_{0}P_{1}\right]+iE\left[Q_{1}P_{0}-Q_{0}P_{1}\right]=-i/2.$$

Thus, we have two constraints on these random variables

(15) $$E\left[Q_{0}Q_{1}+P_{0}P_{1}\right]=0,\;E\left[Q_{0}P_{1}-Q_{1}P_{0}\right]=1/2.$$

**Example** **1.** *Let A,B be independent real random variables with E[A]=E[B]=0, $E\left[A^{2}\right]=E\left[B^{2}\right]=1$, and, in particular, E[AB]=0. Set*

(16) $$X_{0}=\left(A+iB\right)/2,\;X_{1}=\left(-B+iA\right)/2.$$

*Then,*

$$E\left[X_{0}\bar{X_{1}}\right]=\left(1/4\right)E\left[\left(A+iB\right)\left(-B-iA\right)\right]=-i\left(1/4\right)E\left[A^{2}+B^{2}\right]=-i/2$$

*and*

$$E\left[X_{0}\bar{X_{0}}\right]=\left(1/4\right)E\left[A^{2}+B^{2}\right]=1/2=E\left[X_{1}\bar{X_{1}}\right].$$

*So, we reproduced the density operator ρ.*

Now we consider the real representation of complex random variables—the phase space pictures of them. Define $X_{0}=\left(\begin{array}{c} Q_{0}\\ P_{0} \end{array}\right)=\frac{1}{2}\left(\begin{array}{c} A\\ B \end{array}\right),\;X_{1}=\left(\begin{array}{c} Q_{1}\\ P_{1} \end{array}\right)=\frac{1}{2}\left(\begin{array}{c} -B\\ A \end{array}\right).$ Equivalently, $X_{1}=RX_{0}$, where $R=\left(\begin{array}{cc} 0 & -1\\ 1 & 0 \end{array}\right),$ (a $90^{\circ }$ rotation/symplectic matrix).

**Joint vector and block covariance matrix.** Form the joint column vector

(17) $$X=\left(\begin{array}{c} X_{0}\\ X_{1} \end{array}\right)=\left(\begin{array}{c} Q_{0}\\ P_{0}\\ Q_{1}\\ P_{1} \end{array}\right).$$

Its covariance (second-moment, since means are zero) is the 4×4 block matrix $\Sigma \;=\;E\left[\;XX^{T}\;\right]=\left(\begin{array}{cc} E[X_{0}X_{0}^{T}] & E[X_{0}X_{1}^{T}]\\ E[X_{1}X_{0}^{T}] & E[X_{1}X_{1}^{T}] \end{array}\right).$ Compute each block using independence and unit variances: $E\left[X_{0}X_{0}^{T}\right]=\frac{1}{4}\;I_{2},E\left[X_{1}X_{1}^{T}\right]=\frac{1}{4}\;I_{2},$ and the cross-block $E\left[X_{0}X_{1}^{T}\right]=\frac{1}{4}\left(\begin{array}{cc} 0 & 1\\ -1 & 0 \end{array}\right).$ Denote the canonical antisymmetric matrix by $\Omega =\left(\begin{array}{cc} 0 & 1\\ -1 & 0 \end{array}\right).$ (We remark that $\Omega^{2}=-I_{2}.)$. Then, the cross-blocks compactly read $E\left[X_{0}X_{1}^{T}\right]=\frac{1}{4}\;\Omega ,\;E\left[X_{1}X_{0}^{T}\right]={\left(E[X_{0}X_{1}^{T}]\right)}^{T}=-\frac{1}{4}\;\Omega .$ Hence, the full covariance matrix is

(18) $$\begin{array}{c} \;\Sigma \;=\;\frac{1}{4}\left(\begin{array}{cc} I_{2} & \Omega \\ -\Omega & I_{2} \end{array}\right).\; \end{array}$$

**Symplectic interpretation:** The antisymmetric block Ω encodes the canonical symplectic structure on the two-dimensional phase-space (Q,P). Our construction places the two vectors $X_{0}$ and $X_{1}$ in a relative orientation given by that symplectic form: $X_{1}$ is the symplectic (quarter-turn) partner of $X_{0}$.

We can use any four-dimensional random vector *X* (see (17)), with covariance matrix Σ, (see (18)), to generate the superposition (13) with the phase ϕ=π/2. For example, we can use the Gaussian random variable X∼N(0,Σ).

At the level of oscillatory cognition, we have two circuits which generate the complex random variables $Z_{0}=Q_{0}+iP_{0}$ and $Z_{1}=Q_{1}+iP_{1}$, where $Q_{0},Q_{1}$ are electric components and $P_{0},P_{1}$ are magnetic components of generated electromagnetic signals. These electromagnetic signals are coupled via rotation on the angle ϕ=π/2.

### 3.3. Arbitrary Phase ϕ

Now consider the superposition

(19) $$|\psi \rangle =[|0\rangle +e^{i\phi }\left|1\rangle \right]/\sqrt{2},\;\phi \in \left[0,2\pi \right).$$

Consider two classical complex random variables $X_{0},X_{1}$ satisfying $E\left[X_{0}\right]=E\left[X_{1}\right]=0,E[|X_{0}{|}^{2}]=E[|X_{1}{|}^{2}]=\frac{1}{2},$ $E\left[X_{0}\bar{X_{1}}\right]=\frac{e^{-i\phi }}{2}.$ A simple construction is $X_{0}=\frac{1}{\sqrt{2}}Z,$ $X_{1}=e^{i\phi }X_{0}=\frac{e^{i\phi }}{\sqrt{2}}Z,$ where *Z* is any complex random variable with $E\left[Z\right]=0,E[|Z{|}^{2}]=1$, e.g., a standard complex Gaussian Z=U+iV with U,V∼N(0,1) independent.

**Real Quadrature Decomposition** Define $X_{0}=Q_{0}+iP_{0},\;X_{1}=Q_{1}+iP_{1}.$ Then, the quadratures are related by

(20) $$Q_{1}=\cos\phi \;Q_{0}-\sin\phi \;P_{0},\;P_{1}=\sin\phi \;Q_{0}+\cos\phi \;P_{0}.$$

**4 × 4 Real Covariance Matrix** Let $X={\left(Q_{0},P_{0},Q_{1},P_{1}\right)}^{T}$. The covariance matrix $\Sigma =E[XX^{T}]$ is then $\Sigma =\frac{1}{2}\left(\begin{array}{cccc} 1 & 0 & \cos\phi & -\sin\phi \\ 0 & 1 & \sin\phi & \cos\phi \\ \cos\phi & \sin\phi & 1 & 0\\ -\sin\phi & \cos\phi & 0 & 1 \end{array}\right).$ **Verification of Required Moments.**

(21) $$\begin{array}{cccc} E[Q_{0}^{2}+P_{0}^{2}] & =\frac{1}{2},\;\; & \;\;E[Q_{1}^{2}+P_{1}^{2}] & =\frac{1}{2}, \end{array}$$

(22) $$\begin{array}{ccccc} E[Q_{0}Q_{1}+P_{0}P_{1}] & =\frac{\cos\phi }{2}, & & E[Q_{0}P_{1}-Q_{1}P_{0}] & =\frac{\sin\phi }{2}, \end{array}$$

which is exactly consistent with the complex correlation $E\left[X_{0}\bar{X_{1}}\right]=e^{-i\phi }/2$.

## 4. Random Complex Vector with Covariance Equal to a Pure State Density Matrix

Consider now an arbitrary pure quantum state

(23) $$|\psi \rangle =\sum_{k=0}^{n-1}c_{k}\;|k\rangle ,$$

with complex coefficients $c_{k}\in C$, normalized so that $\sum_{k}{\left|c_{k}\right|}^{2}=1$. The corresponding density matrix is $\rho =|\psi \rangle \langle \psi |,\;\rho_{kj}=c_{k}c_{j}^{*}.$ Let $Z=\left(Z_{0},\ldots ,Z_{n-1}\right)\in C^{n}$ be a random vector with a zero mean and a covariance equal to ρ: $C=\mathrm{Cov}\left[Z\right]=E\left[ZZ^{†}\right]=\rho .$

Since ρ is a pure-state density matrix, it is rank-1 with a single nonzero eigenvalue λ=1 and eigenvector |ψ〉. Therefore, the random vector *Z* must lie entirely in the direction of |ψ〉:

(24) $$\begin{array}{c} Z=\xi \;|\psi \rangle , \end{array}$$

where ξ is a complex scalar random variable.

The covariance condition gives $C=E\left[ZZ^{†}\right]={E[|\xi |}^{2}]\;|\psi \rangle \langle \psi |=\rho =|\psi \rangle \langle \psi |,$ so $E[|\xi {|}^{2}]=1.$ Additionally, since E[Z]=0, we have E[ξ]=0.

### Conclusions

The random vector *Z* is always proportional to the pure state vector |ψ〉, with the proportionality factor ξ being a zero-mean complex scalar of unit variance:

(25) $$Z=\xi \;|\psi \rangle ,\;E\left[\xi \right]={0,\;E[|\xi |}^{2}]=1.$$

All randomness of *Z* occurs in the direction of |ψ〉, with no components in the orthogonal subspace.

Pure state |ψ〉 is generated by an array of neuronal circuits, and circuit *k* generates the complex random variable $Z_{k}=Z_{k}\left(\omega \right)=c_{k}\xi \left(\omega \right).$

We can connect phases $\left(\phi_{k}\right)$ in the quantum-like state |ψ〉 with phases $\left(\theta_{km}\right)$ of the matrix elements $c_{km}$ of C.

## 5. Concluding Remarks

Pure states play a central role in QLM and, in particular, in QCDM, where they represent superpositions of cognitive alternatives. In existing models, the phases of these states have typically been introduced at a phenomenological level, treated merely as additional free parameters. However, the problem of interpreting these phases remains crucial for the foundational justification of QLM and, especially, for establishing a coherent bridge between QCDM and neurocognitive mechanisms.

Building on the bridging framework developed in [6] (please also see [23,24]), we have proposed an interpretation in which the phases arise from the activity of networks of neuronal circuits. This connection provides a more principled grounding for the role of phases in quantum-like superpositions and opens a pathway toward integrating QCDM with classical neurocognitive models.

## Data Availability

No new data were created or analyzed in this study. Data sharing is not applicable to this article.
